# Association of sphenoid sinus pneumatization patterns, Onodi cells, and rostrum pneumatization with natural ostium location: A cross-sectional CT study

**DOI:** 10.1097/MD.0000000000049344

**Published:** 2026-06-19

**Authors:** Minh Quang Nguyen, Minh Phuong Tang, Hung Tien Nguyen, Huong Thi Tran, Trinh To Tran, Nguyen Binh Minh Hoang Tran

**Affiliations:** a Department of Otolaryngology, Faculty of Medicine, University of Health Sciences, Vietnam National University, Ho Chi Minh City, Vietnam; b International PhD Program in Medicine, College of Medicine, Taipei Medical University, Taipei, Taiwan; c Department of General Surgery, Tra Vinh General Hospital, Vinh Long Province, Vietnam; d Department of Family Medicine, Faculty of Medicine, Pham Ngoc Thach University of Medicine, Ho Chi Minh City, Vietnam; e Department of Pharmacy, Lac Hong University, Dong Nai Province, Vietnam; f Office of Personnel and Administration, University of Health Sciences, Vietnam National University, Ho Chi Minh City, Vietnam; g Department of Rehabilitation, Ho Chi Minh City Hospital for Rehabilitation and Professional Diseases, Ho Chi Minh City, Vietnam.

**Keywords:** Onodi cell, sphenoid ostium, sphenoid rostrum pneumatization, sphenoid sinus pneumatization

## Abstract

Endoscopic management of sphenoid sinus pathologies requires a meticulous understanding of the regional anatomy to avoid injury to adjacent critical structures. Since the natural sphenoid ostium provides a consistent and preferred entry point, clarifying its position relative to various pneumatization patterns is essential for successful surgical planning. This study aimed to investigate the relationship between the location of the sphenoid sinus ostium and sphenoid sinus pneumatization patterns, including the presence of Onodi cells and pneumatization of the sphenoid rostrum, using computed tomography (CT). We conducted a cross-sectional descriptive study of paranasal sinus CT scans of 181 adult patients (362 sphenoid sinuses, both sides analyzed separately) at a tertiary ear–nose–throat hospital. Sphenoid sinus pneumatization was classified in the sagittal plane as presellar, sellar, or postsellar, and in the coronal plane as Type 1 (previdian), Type 2 (intercanal), or Type 3 (post rotundum) based on the degree of lateral extension. The presence of Onodi cells and pneumatization of the sphenoid rostrum (anterior sphenoid beak) were recorded. We measured the horizontal and vertical diameters of the sphenoid ostium, along with distances to adjacent landmarks: D1, superior border of the ostium to the skull base; D2, inferior ostium to the superior border of the choana; D3, medial ostium to the nasal septum; D4, lateral ostium to the orbital wall; and D5, sphenoid sinus roof to floor (sinus height). The study found that all sphenoid sinuses had a single natural ostium, with mean horizontal and vertical diameters of 1.25 mm and 1.82 mm, respectively. Onodi cells (141/362 sinuses, 39%) were associated with a significantly greater ostium-to-choana distance (D2: 11.77 vs 10.00 mm, *P* <.001). A pneumatized sphenoid rostrum (53%) was the strongest predictor of non-medial ostium placement (OR = 15.86, *P* <.001), while extensive lateral pneumatization (Type 3) was associated with medial ostium displacement (OR = 0.32, *P* = .002). Sphenoid sinus pneumatization patterns and anatomical variations, such as pneumatized sphenoid rostrum and Onodi cells, were significantly associated with the location of the ostium. Preoperative CT evaluation of these variations may assist surgeons in anticipating ostium positioning and planning safer endoscopic approaches.

## 1. Introduction

The sphenoid sinus is associated with complex pathologies requiring endoscopic surgical management, which remains challenging due to the proximity of critical neurovascular structures.^[1]^ Endoscopic approaches to the sphenoid sinus and skull base carry substantial risks of complications.^[2,3]^ Among the multiple surgical corridors, access via the natural ostium is often preferred because it minimizes the risk of injury to surrounding structures.^[4,5]^ Therefore, a comprehensive understanding of the ostium position relative to the nasal septum, skull base, and choana is essential for safe surgical planning.

Numerous studies have examined the effect of sphenoid sinus pneumatization on surrounding anatomical structures. For example, extensive sphenoid pneumatization can lead to protrusion or dehiscence of the optic nerve or internal carotid artery canals,^[6]^ underscoring the importance of recognizing anatomical variations in this region.^[7]^ However, previous studies have primarily examined the effect of pneumatization on neurovascular structures or have reported general ostium measurements without analyzing their relationship to specific pneumatization subtypes.^[8]^ While recent investigations by Doubi et al and Kim et al have provided valuable data on ostium location,^[9,10]^ the combined influence of AP pneumatization, lateral pneumatization, Onodi cells, and rostrum pneumatization on ostium position has not been systematically evaluated in a single cohort.

In this study, we sought to locate the sphenoid sinus ostium (SO) on computed tomography (CT) scans and analyze its relationship with the AP pneumatization type of the sphenoid sinus, the degree of lateral (transverse) pneumatization of the sphenoid sinus, the presence or absence of an Onodi cell, and the presence or absence of a pneumatized sphenoid rostrum (PSR). We hypothesized that Onodi cells would alter the vertical position of the ostium, while pneumatization of the sphenoid rostrum and the degree of lateral pneumatization would influence its horizontal position on the anterior sphenoid face.

## 2. Materials and methods

### 2.1. Study design and population

We performed a descriptive cross-sectional study of paranasal sinus CT scans. The study sample consisted of 181 consecutive patients aged ≥ 18 years who underwent sinus multislice CT at the Ho Chi Minh City Ear–Nose–Throat Hospital between September 2022 and December 2022. This yielded a total of 362 sphenoid sinuses for analysis (each patient contributing 2 sphenoid sinuses). Only patients with fully developed paranasal sinuses aged 18 years were included to ensure mature sinus anatomy and avoid developmental variations due to age.

Patients were excluded if they had congenital craniofacial abnormalities; a history of trauma or prior endoscopic surgery involving the posterior ethmoid, sphenoid sinus, or anterior skull base, which could alter the sphenoid anatomy; or significant sphenoid sinus pathology (e.g., tumors or invasive fungal sinusitis) resulting in anatomical distortion of the sphenoid, bony destruction, or new bone formation. The minimum required sample size was calculated using the following formula for estimating a single proportion: N = Z^2^_1-α/2_ × p(1 − p)/ d^2^ with Z_1_₋α/_2_ = 1.96, *P* = .38 (prevalence of Onodi cells reported by Doubi et al.), and d = 0.05, yielding a minimum of 362 sinuses. All eligible consecutive patients during the study period were enrolled, resulting in 181 patients (362 sinuses) meeting this requirement.

### 2.2. Data collection

CT scans were acquired using a 128-slice multidetector CT scanner (Siemens) with standardized parameters: 120 kV, 75 mAs, detector configuration 16 × 0.75 mm, and a slice thickness of 0.6 mm with no interslice gap. Axial images were subsequently reformatted into sagittal and coronal planes using RadiAnt DICOM Viewer (version 2025.2.0.13300; Medixant, Poznań, Poland), with a reconstruction thickness of 0.6 mm. All measurements were performed on bone window settings (W: 2500, L: 3500). The axial dataset encompassed cuts from the level of the hard palate (maxillary tooth apices) to the frontal sinus roof. Sagittal reformats were obtained along a plane through the nasal bridge and the midsella. Coronal reformats were obtained perpendicular to the hard palate, extending from the anterior frontal sinus wall to the clivus. Sections through the anterior clinoid (planum sphenoidale) and optic canal were closely examined for sphenoid configuration.

On each scan, we identified the sphenoid SO and noted its number (single or multiple ostia per sinus) and measured its largest horizontal (transverse) and vertical diameters. We recorded the position of the ostium on the anterior sphenoid wall in 2 dimensions: vertical position (sagittal plane) and horizontal position (coronal plane). To quantify this, we divided the anterior face of the sphenoid sinus into a 3 × 3 grid of equal thirds. Vertically, the anterior wall was divided into upper, middle, and lower thirds, while horizontally, it was divided into medial (closest to the nasal septum), middle, and lateral thirds. Each ostium was classified according to which third of the anterior wall it occupied, vertically and horizontally.

**Figure 1.**
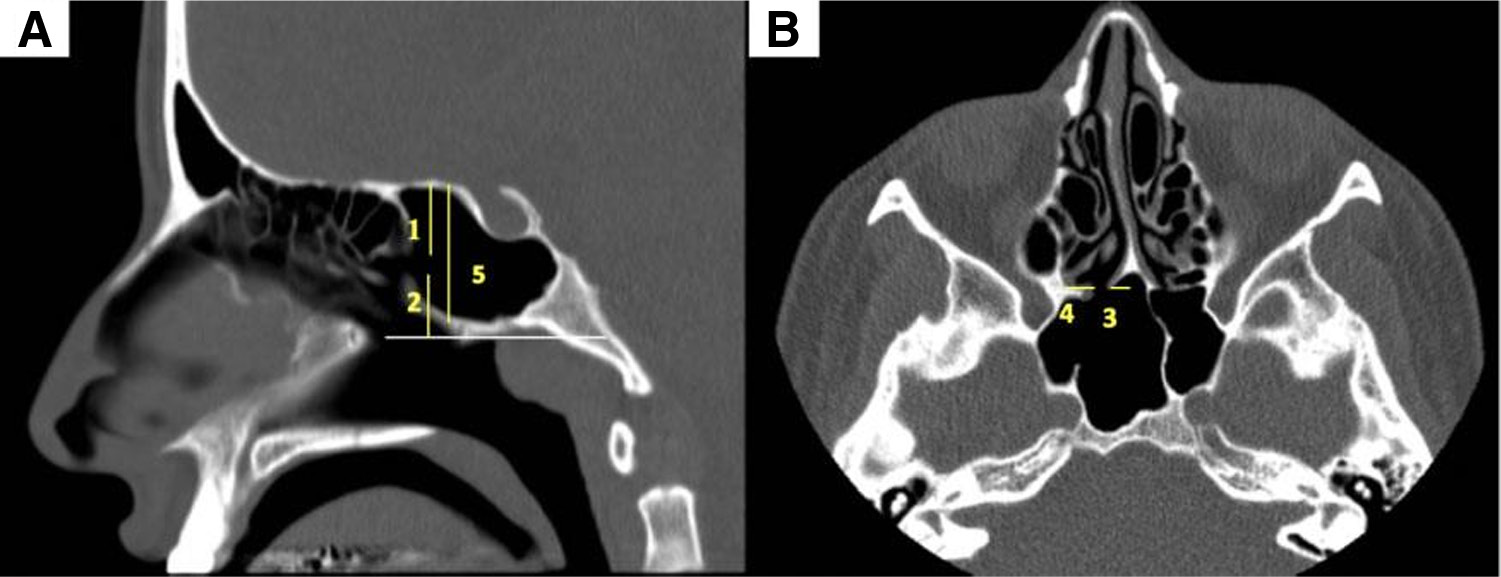
Method of measuring distances from the sphenoid sinus ostium to adjacent anatomical landmarks. D1, from the superior ostium to the skull base; D2, from the inferior ostium to the superior border of the choana; D3, from the medial ostium to the nasal septum; D4, from the lateral ostium to the orbital wall; D5, sphenoid sinus height (distance from the roof to the floor of the sinus).

We also measured key distances from the sphenoid ostium to surrounding anatomical landmarks (on reformatted planes), defined as follows and illustrated in Figure 1:

D1: Distance from the superior border of the SO to the skull base (planum sphenoidale).

D2: Distance from the inferior border of the SO to the superior border of the choana (i.e., to the roof of the nasopharynx at the level of the sphenoid floor).

D3: Distance from the medial border of the SO to the nasal septum.

D4: Distance from the lateral border of the SO to the lamina papyracea (orbital wall).

D5: Distance from the sphenoid sinus roof to the sphenoid sinus floor (i.e., height of the sphenoid sinus).

For each sphenoid sinus, we assessed the pneumatization pattern in 2 directions: AP and transverse (lateral). AP pneumatization was categorized on midsagittal images as either presellar, sellar, or postsellar type, according to the extent of pneumatization relative to the sella turcica. In presellar type, the sinus does not extend beyond the anterior wall of the sella; in sellar type, the sinus extends beneath the sella turcica; and in postsellar type, the sinus extends beyond the dorsum sellae into the clival region. Transverse (horizontal) pneumatization was categorized on coronal images into 3 types based on lateral extension of the sphenoid air cavity: Type 1 (previdian), limited pneumatization (sinus remains medial and does not reach the vidian canal region laterally); Type 2 (intercanal), moderate lateral pneumatization (extending to between the neurovascular canals, reaching the level of the vidian canal or optic nerve canal); and Type 3 (postrotundum), extensive lateral pneumatization (expanding laterally beyond the foramen rotundum into the greater wing of sphenoid). PSR was defined as aeration of the sphenoid bone protrusion at the anterior midline (sphenoid beak) such that an air cavity was present within the rostrum separate from the main sinus cavity. The Onodi cell (sphenoethmoidal cell) was identified on CT when a posterior ethmoid air cell extended superolateral to the sphenoid sinus, with the optic nerve canal bulging into the cell and the cell wall interposed between the sphenoid sinus and optic nerve. Multiplanar reconstructions were carefully reviewed to identify Onodi cells; an Onodi cell was confirmed according to the criteria described by Wada et al., based on the relationship of the ethmoid air cell to the optic nerve canal, sphenoid sinus, and sella. Each sinus was noted as having an Onodi cell present or absent.

All measurements were performed using the RadiAnt DICOM viewer measuring tools. Two experienced ear–nose–throat surgeons independently reviewed a randomly selected subset of 30 CT scans (60 sphenoid sinuses), including ostium identification, D1–D5 measurements, and classification of pneumatization patterns and Onodi cells. Discrepancies were resolved by consensus with reference to multiplanar reconstructions. High concordance was observed, and the remaining scans were measured by the primary investigator using the same standardized protocol. Measurements were reported in millimeters (mm).

### 2.3. Data analysis

Data were analyzed using SPSS version 21 (IBM, Armonk), Microsoft Excel 2016, and R version 4.x for supplementary analyses. Descriptive statistics (mean, SD, minimum, and maximum) were computed for continuous variables such as distances (D1–D5) and ostium diameters. Categorical variables (e.g., pneumatization type, ostium position categories, and presence of Onodi cells) were summarized as frequencies and percentages. The normality of continuous variables was assessed using the Shapiro–Wilk test and visual inspection of histograms; homogeneity of variances was verified using Levene test prior to parametric analyses. We used an independent t-test to compare the mean values of continuous variables between 2 groups (e.g., male vs female, left vs right side) and one-way ANOVA for comparisons across more than 2 groups (e.g., comparing distances D1–D5 among presellar, sellar, and postsellar groups). The chi-square (χ^2^) test was used to assess associations between categorical variables (e.g., presence of Onodi cells vs ostium position categories). Pearson correlation was used to examine the linear relationships between the sphenoid sinus height (D5) and the vertical ostium distances (D1, D2). To account for the within-subject correlation of bilateral sinuses, the Intraclass Correlation Coefficient (ICC) was calculated for each measurement (D1–D5). Variables with ICC ≥ 0.50 were re-analyzed using Linear Mixed-Effects Models with patient as a random intercept; variables with ICC <0.50 were treated as independent observations. Finally, a multinomial logistic regression analysis was performed to identify independent predictors of the horizontal ostium location (medial, middle, or lateral third) on the anterior sphenoid wall, with the medial third as the reference category. Covariates included the sphenoid lateral pneumatization type and the presence of a pneumatized rostrum, which were significant factors in univariate analysis. Age and sex were evaluated in univariate analyses but showed no significant association with the horizontal ostium position and were therefore excluded from the final model; a supplementary Likelihood Ratio test confirmed that their inclusion did not improve model fit (*P* = .703). Multicollinearity among predictors was assessed using variance inflation factors and Cramér V. Model performance was evaluated using the Nagelkerke R^2^, Likelihood Ratio test, and overall classification accuracy. Results with *P* <.001 (two-tailed) were considered statistically significant. This conservative threshold was adopted a priori to account for multiple comparisons across anatomical subgroups, thereby reducing the risk of Type I error without requiring formal correction procedures (e.g., Bonferroni), which may be overly restrictive in exploratory anatomical studies.

### 2.4. Ethical considerations

All included CT scans were taken for diagnostic purposes (such as evaluation of sinus symptoms or preoperative planning) and de-identified for analysis. This study followed the Declaration of Helsinki and was approved by the institutional ethics board (approval code: 594/TĐHYKPNT-HĐĐĐ, approval date: February 15, 2022). Informed consent was obtained from all participants for the use of their imaging data in the study.

## 3. Results

The study included a total of 181 patients (362 sphenoid sinuses) with a mean age of 45.6 ± 13.9 years. There was a female predominance (60.2%), and complete data were available for all participants. Age, sex, and laterality did not have a significant effect on the measured anatomical parameters (all *P* > .05). The side distribution was balanced (181 right, 181 left), with no significant differences in D1–D5 or ostium position between sides. ICC analysis revealed good bilateral agreement for D1 (0.766) and D2 (0.754), moderate agreement for D3 (0.520) and D4 (0.527), and poor agreement for D5 (0.088). A solitary natural ostium was identified in all cases, measuring 1.25 ± 0.59 mm transversely and 1.82 ± 1.01 mm vertically on average. Spatially, the ostium favored the medial third (57.7%) of the anterior face and the middle vertical third (73.2%). The detailed distances to the key landmarks (D1–D5), along with LMM-derived estimates for D1–D4, are provided in Table 1.

**Table 1:** Measurements of distances from the sphenoid sinus ostium to adjacent anatomical landmarks (in millimeters).

Distance and landmark	Minimum (mm)	Maximum (mm)	Mean ± SD (mm)	Estimated Mean ± SE (mm)	95% CI
D1: Ostium top to skull base	1.73	20.80	9.84 ± 3.11	9.84 ± 0.22*	9.41–10.27
D2: Ostium bottom to choana	1.64	25.50	10.69 ± 3.52	10.69 ± 0.25*	10.21–11.18
D3: Ostium medial to septum	0.23	9.93	4.01 ± 1.51	4.01 ± 0.10*	3.82–4.20
D4: Ostium lateral to orbit	2.65	16.80	9.70 ± 2.40	9.70 ± 0.16*	9.40–10.01
D5: Sinus height (roof to floor)	4.22	30.00	18.87 ± 4.12	18.87 ± 0.22**	18.44–19.30

CI = confidence interval, LMM = Linear Mixed-Effects Models, SD = standard deviation, SE = standard error.

* Estimated marginal means, SE, and 95% CIs for D1, D2, D3, and D4 were calculated using LMM with the patient as a random effect to adjust for the within-subject correlation of bilateral sinuses (intraclass correlation coefficient >0.5).

** Based on a poor intraclass correlation coefficient indicating biological independence between the left and right sides, D5 was analyzed using standard statistical methods assuming 362 independent measurements.

### 3.1. Onodi cells and ostium relationship

**Figure 2.**
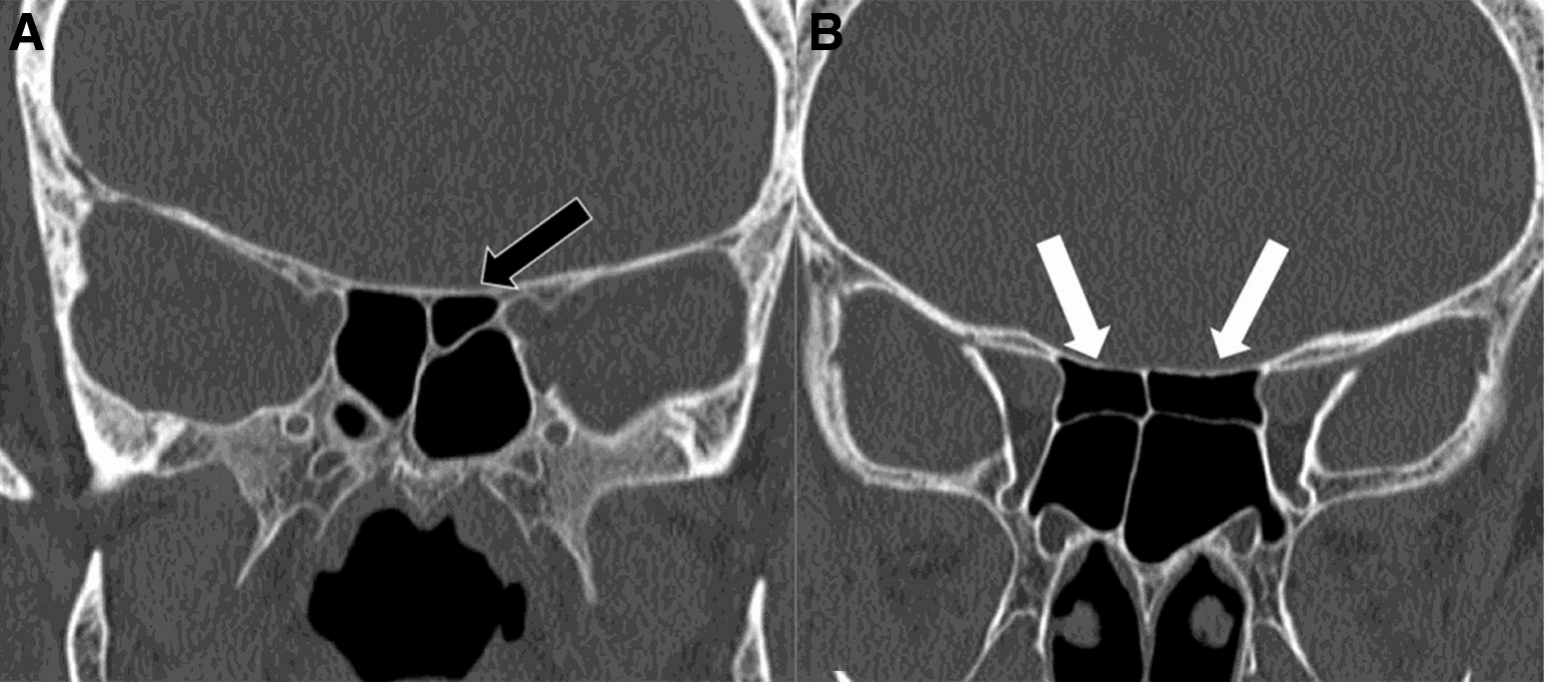
Coronal CT scan showing an Onodi cell (sphenoethmoidal cell, indicated by the arrow) superior and lateral to the sphenoid sinus. The presence of such a cell can alter the surrounding anatomy and is associated with a higher sphenoid ostium position.

Onodi cells were identified in 39% (141/362) of the sinuses, exhibiting a mean vertical height of 5.41 ± 2.7 mm. Statistical analysis revealed no significant predilection regarding sex or laterality. The mean D2 was significantly greater in the Onodi group (11.77 ± 3.60 mm) than in the non-Onodi group (10.00 ± 3.29 mm; *P* <.001). The D1 did not differ significantly between groups (Onodi: 10.28 ± 2.59 mm vs non-Onodi: 9.56 ± 3.38 mm; *P* = .033). Onodi cell presence was associated with predominant ostium localization in the middle vertical third of the anterior face (Fig. 2).

### 3.2. Pneumatized sphenoid rostrum and ostium relationship

**Figure 3.**
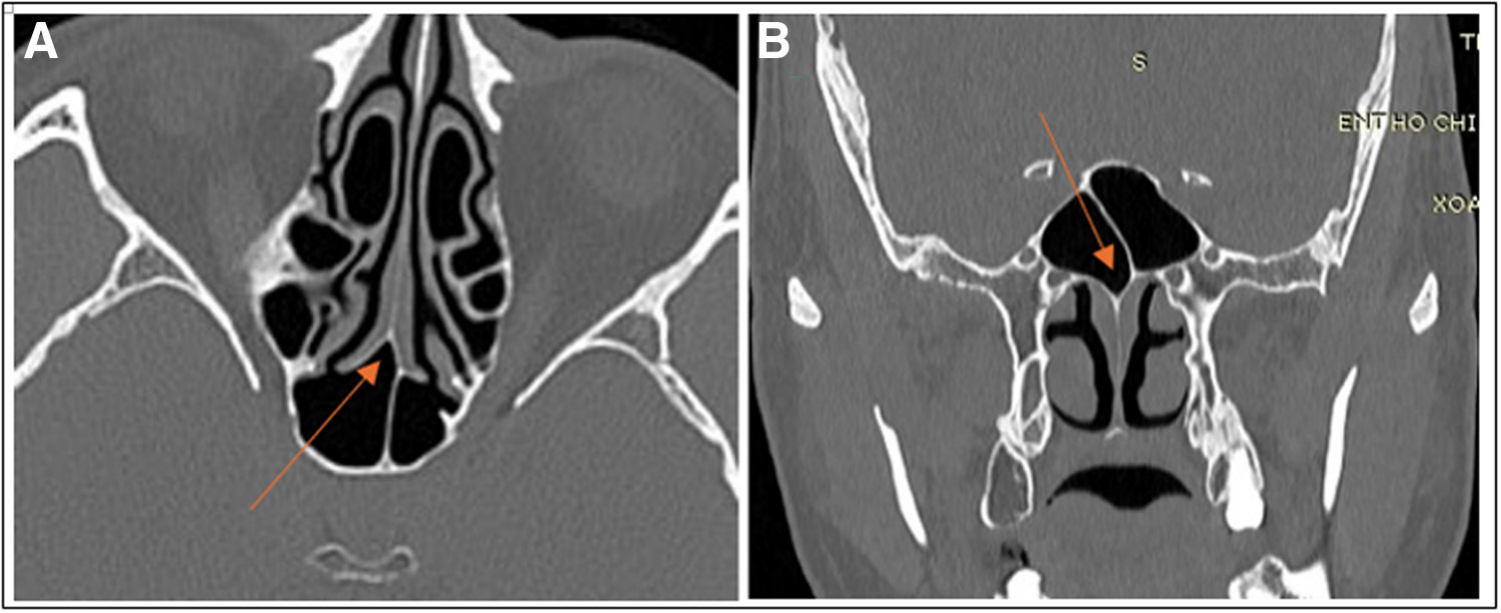
Pneumatization of the sphenoid rostrum observed on axial and coronal CT images (arrow). CT = computed tomography.

A PSR was documented in 53% of cases, exhibiting a mean depth of 5.86 ± 1.4 mm with no significant sex predilection (Fig. 3). The mean D3 was significantly greater in the PSR group (4.58 ± 1.56 mm) than in the non-PSR group (3.36 ± 1.14 mm; *P* <.001), while D4 was significantly smaller (PSR: 9.17 ± 2.59 mm vs non-PSR: 10.31 ± 2.02 mm; *P* <.001). PSR presence was associated with ostium localization in the middle or lateral thirds of the anterior face, whereas non-pneumatized rostrums were associated with medial ostium placement (*P* <.001).

### 3.3. Sphenoid sinus pneumatization (anteroposterior and lateral) and ostium relationship

#### 3.3.1. *Anteroposterior pneumatization types*:

**Figure 4.**
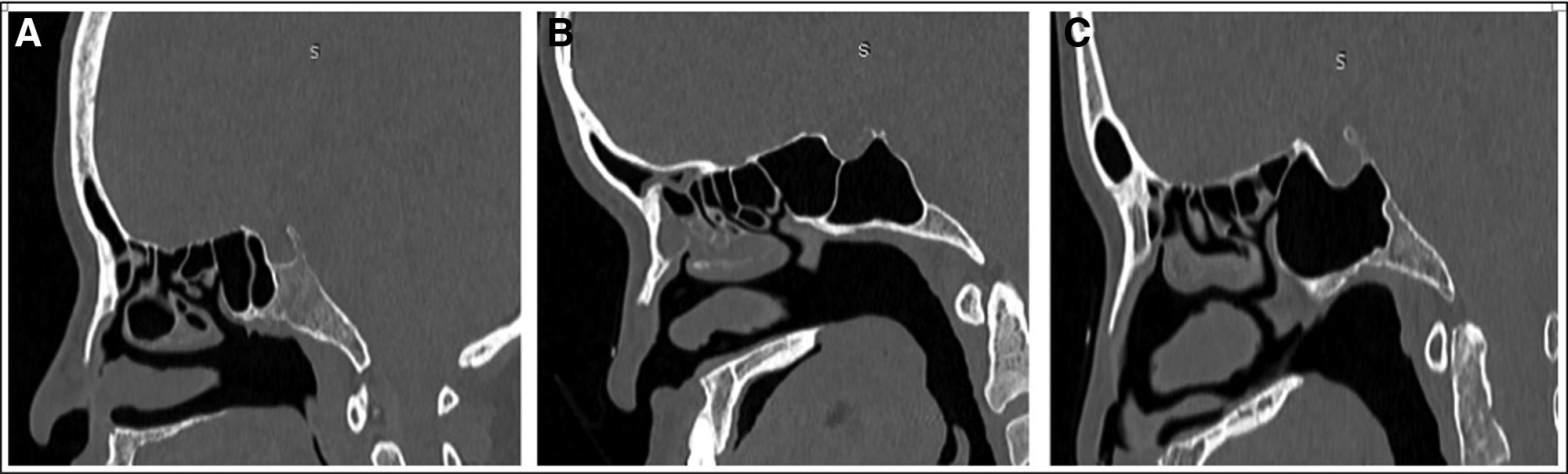
Types of sphenoid sinus pneumatization in the anterior–posterior direction: (A) presellar type, (B) sellar type, and (C) postsellar type.

Postsellar configuration was predominant in the cohort (52.8%), followed by sellar (40.9%) and presellar (6.4%) (Fig. 4). ANOVA showed no significant differences in D1 (*P* = .211) or D2 (*P* = .264) across presellar, sellar, and postsellar groups. Pearson analysis revealed a modest inverse correlation between sinus height (D5) and vertical ostium distances (D1, D2).

#### 3.3.2. Lateral (transverse) pneumatization types:

**Figure 5.**
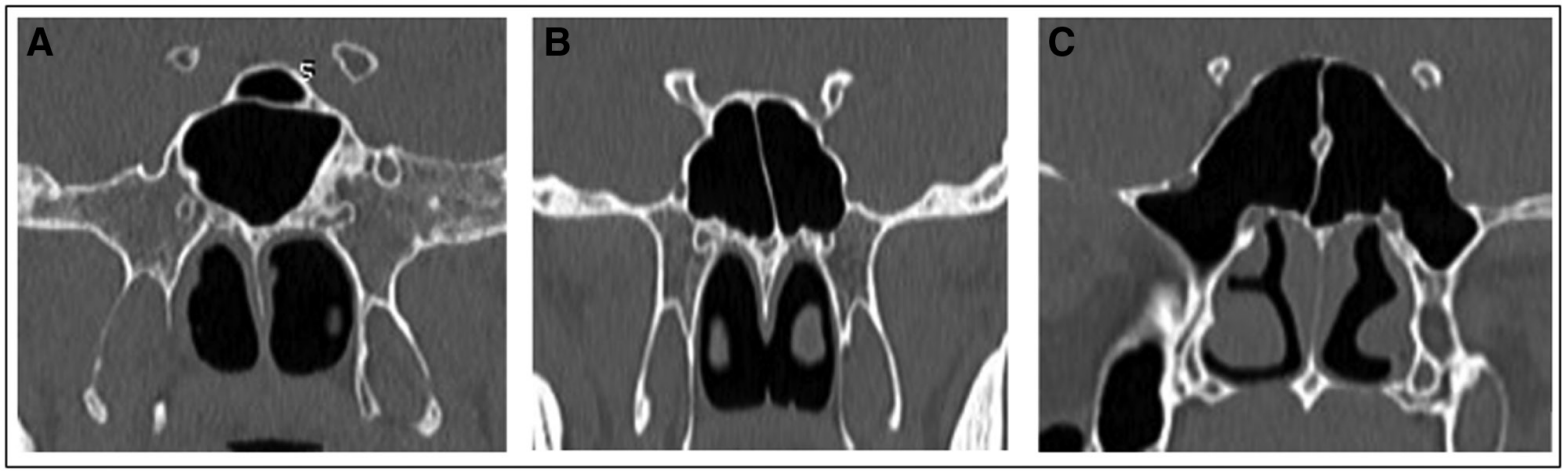
Types of sphenoid sinus pneumatization in the transverse (lateral) direction, from left to right: narrow pneumatization (Type 1), intermediate pneumatization (Type 2), and extensive lateral pneumatization (Type 3).

Lateral pneumatization types significantly influenced horizontal ostium topography (*P* <.001). Type 3 (postrotundum) was the most frequent (50.6%), followed by Type 2 (30.1%) and Type 1 (19.3%) (Fig. 5). The mean D4 differed significantly across groups (ANOVA: F = 28.4, *P* <.001): Type 1, 8.41 ± 2.13 mm; Type 2, 9.12 ± 2.02 mm; Type 3, 10.55 ± 2.40 mm.

Multinomial logistic regression identified both a pneumatized rostrum and lateral pneumatization type as independent predictors of horizontal ostium location (*P* <.001; Nagelkerke R^2^ = 0.405; overall classification accuracy = 75.1%). No multicollinearity was detected among the predictors (all variance inflation factors <2; Cramér’s V = 0.035). For the comparison of middle third versus medial third (reference), PSR was the strongest predictor of non-medial ostium placement (OR = 15.86, 95% CI: 8.95–28.11, *P* <.001), while Type 3 lateral pneumatization was associated with decreased odds of middle-third placement (OR = 0.32, 95% CI: 0.15–0.65, *P* = .002), consistent with medial displacement. Type 2 lateral pneumatization showed a similar but nonsignificant trend (OR = 0.56, 95% CI: 0.26–1.21, *P* = .141). The comparison of the lateral third versus the medial third could not be reliably estimated due to the small number of lateral-third cases (n = 8).

## 4. Discussion

Our study focused exclusively on adults to eliminate developmental variability in sinus pneumatization. The demographic profile (mean age of approximately 46 years, with female predominance) mirrors that of previous cohorts in CT-based studies, ensuring that our findings are broadly representative.^[4,11–14]^ Consistent with the findings of Kaplanoglu et al., we observed no significant sexual dimorphism in the sphenoid measurements, justifying a unified analysis without sex stratification.^[12]^

A single natural ostium was identified in 100% of the patients. Although anatomical variations such as multiple, accessory, or absent ostia have been documented in previous cadaveric and imaging literature, our data suggest that these are exceptional anomalies in the general population.^[13,15–17]^ Regarding dimensions, we observed mean diameters of 1.25 mm (width) and 1.82 mm (height). These values are notably smaller than the 3 to 4 mm range reported in cohorts by Doubi et al and Jaworek-Troć et al.^[4,9]^ This discrepancy may be attributed to several factors: first, differences in craniofacial morphology between Asian and European populations, as sphenoid sinus dimensions have been shown to vary across ethnic groups; second, differences in measurement methodology, as some studies measured the ostium after mucosal retraction in cadaveric specimens or used different CT window settings and thresholds for defining the bony ostium margins. Clinically, this small aperture size underscores the challenge of endoscopic localization, necessitating reliance on consistent anatomical landmarks such as the superior turbinate. The lack of significant sexual or lateral dimorphism suggested that a small ostium is a consistent feature across the adult population.

Defining the precise topography of the ostium is critical for safe transsphenoidal access.^[3,17–19]^ Vertically, our findings define a clear surgical corridor: the ostium typically lies approximately 10 mm below the planum sphenoidale (D1) and an average of 10.7 mm above the choana (D2). This substantial distance from the skull base aligns with that reported by Halawi et al., suggesting a safety margin to prevent inadvertent intracranial injury.^[11]^ Similarly, we found that the suprachoanal height supports the trajectory planning principles emphasized by Twigg et al.^[13,20]^

Horizontally, the ostium demonstrates a strong medial predilection. We measured an average distance of approximately 4 mm from the nasal septum (D3). Although slightly wider than the 2 mm reported by Campero et al.,^[21]^ this supports the observation of Gupta et al that the ostium consistently resides medial to the midline of the sinus.^[17]^ Indeed, 57.7% of our cases were located in the medial third, supporting the use of the superior turbinate attachment as a reliable surgical landmark. Furthermore, we noted an inverse correlation between the total sinus height and ostium position; that is, taller, vertically extensive sinuses tended to feature inferiorly displaced ostia. This suggests that the ostium may be positioned slightly lower in highly pneumatized sphenoids, a variation partially hypothesized by Halawi et al.^[11]^

### 4.1. Influence of Onodi cells

The surgical significance of Onodi cells lies in their close relationship with the optic nerve.^[6]^ While the reported prevalence varies widely – from 8% to 24% in radiological series to 60% in cadaveric studies – our multiplanar analysis based on the criteria of Wada yielded a prevalence of 39%.^[6]^ This relatively high detection rate underscores the utility of sagittal and coronal reviews in identifying cells potentially missed by single-planar imaging.^[22]^ Crucially, the Onodi cells significantly influenced the vertical topography of the ostium. We observed a marked increase in the ostium-to-choana distance (D2), whereas the distance to the skull base (D1) remained constant. This differential suggests that the expansion of the Onodi cells displaces the choanal roof inferiorly, effectively creating a higher ostium relative to the nasopharyngeal floor. This mechanism aligns with that reported by Doubi et al., who similarly noted that posterior ethmoid cells alter the apparent vertical coordinates of the sphenoid ostium.^[4]^ Consequently, when an Onodi cell is identified preoperatively, surgeons should anticipate a more superiorly located ostium relative to the choana despite its stable relationship with the skull base.

### 4.2. Influence of sphenoid rostrum pneumatization

A PSR was identified in 53% of cases, slightly lower than the approximately 60% reported by Jaworek-Troć et al and Doubi et al.,.^[4,9]^ This difference may reflect variations in the CT criteria used to define rostrum pneumatization, as there is currently no universally standardized threshold for this classification. Additionally, ethnic differences in sphenoid bone morphology may contribute to prevalence variation across populations. Significantly, PSR was strongly associated with lateral ostium displacement. The observed lateral displacement may be explained by expansion of the rostrum extending the medial sinus boundary, as supported by the increased septum-to-ostium distance (D3) and reduced orbit-to-ostium distance (D4). This finding provides quantitative support for the observations of Doubi et al and aligns with Kim et al., who recently demonstrated via logistic regression that increased rostral width is associated with ostium lateralization.^[4,10]^ Clinically, this suggests a preoperative consideration: identifying a PSR on CT should prompt the surgeon to anticipate a laterally displaced ostium and avoid futile dissection at the typical medial landmark. Furthermore, our multivariate analysis suggested a broader pattern in which the ostium migrates contralaterally to the vector of pneumatization: anterior rostral expansion displaces the ostium laterally, whereas lateral recess expansion shifts it medially.

### 4.3. Influence of sphenoid sinus pneumatization patterns

Regarding the AP extent, the postsellar configuration predominated (52.8%), a distribution comparable to that of Doubi et al but exceeding the rates reported by Halawi et al.^[4,11]^ Critically, while Halawi et al suggested that presellar sinuses feature higher-positioned ostia, our analysis revealed no statistically significant association between AP classification and vertical ostium topography. This discrepancy may be explained by differences in sample composition: our cohort had a lower proportion of presellar sinuses and a larger sample size (n = 362 vs 202), which may have provided greater statistical power to detect or exclude such an association. Our findings align with those of Doubi et al., supporting the conclusion that AP depth alone is not a primary determinant of ostium height (Table 2).^[4,11]^ In contrast, lateral pneumatization was significantly associated with horizontal positioning. Consistent with Doubi et al., we found that extensive lateral pneumatization (Type 3) correlates with medialization of the ostium, whereas confined pneumatization (Type 1) is associated with more lateral placement.^[4]^ This inverse relationship suggests a practical consideration: evidence of pterygoid or greater wing pneumatization on CT should alert the surgeon to a medially retracted ostium that is potentially concealed behind the superior turbinate. Our multivariate analysis is consistent with the observations of Kim et al., suggesting that the ostium position may reflect competing vectors of sinus expansion: rostral width is associated with lateralization, whereas lateral recess expansion is associated with medialization.^[10]^

**Table 2:** Comparison of sphenoid sinus pneumatization patterns (anterior–posterior and lateral) across different studies in the literature versus the present study.

Study	Presellar (%)	Sellar (%)	Postsellar (%)	Type 1/narrow (%)		Type 2/intermediate (%)	Type 3/lateral (%)
Halawi et al, 2015	20.3	51.0	28.7		–	–	–
Doubi et al, 2020	6.8	59.0	34.0		22.3	39.7	38.0
Present study, 2025	6.4	40.9	52.8		19.3	30.1	50.6

Clinically, these findings provide anatomical observations that may assist in transsphenoidal access. An Onodi cell was associated with a vertically elevated ostium relative to the choana, whereas rostral and lateral pneumatization are associated with lateral and medial shifts, respectively. The observed 10-mm vertical clearance from both the skull base and choana suggests a safe surgical corridor, supporting the superior turbinate as a reliable landmark. This study has several limitations. First, bilateral sinuses were analyzed as separate observations. Although supplementary ICC and LMM analyses confirmed minimal impact on descriptive results, the inferential tests were not formally adjusted for clustering. However, given the consistently low *P*-values (<.001) and marginal effect on standard errors, the conclusions are unlikely to be materially affected. Second, interobserver reliability metrics (kappa, ICC) were not formally reported despite consistency review by 2 surgeons. Third, the retrospective single-center design, absence of intraoperative validation, and ethnic homogeneity of the cohort may limit generalizability. Future studies should employ clustered analytical methods and validate these findings across diverse populations with intraoperative correlation.

## 5. Conclusions

Our study demonstrates that sphenoid sinus pneumatization patterns and adjacent anatomical variations are significantly associated with the location of the sphenoid sinus. While the ostium is typically situated mid-height toward the medial side of the anterior sphenoid face, its position varies with specific anatomical factors: Onodi cells were associated with a higher vertical placement; a PSR was associated with lateral displacement; and extensive lateral pneumatization was associated with medial displacement. Notably, AP pneumatization (presellar vs postsellar) did not significantly affect ostium positioning.

Preoperative CT evaluation of these anatomical variations, including the presence of Onodi cells, the degree of pneumatization, and rostrum pneumatization, may provide useful guidance for anticipating ostium location during endoscopic surgery. Incorporating these observations into preoperative planning may contribute to improved surgical orientation and safer approaches to the sphenoid sinus and sellar region.

## Acknowledgments

We would like to express our sincere gratitude to the Department of Diagnostic Imaging, Ho Chi Minh City Ear, Nose and Throat Hospital, and the Faculty of Medicine, University of Health Sciences, Vietnam National University, Ho Chi Minh City, for their support and collaboration during this study.

## Author contributions

**Conceptualization:** Minh Quang Nguyen.

**Data curation:** Minh Quang Nguyen, Nguyen Binh Minh Hoang Tran.

**Formal analysis:** Minh Quang Nguyen, Minh Phuong Tang.

**Investigation:** Minh Phuong Tang, Hung Tien Nguyen.

**Methodology:** Minh Quang Nguyen, Huong Thi Tran, Nguyen Binh Minh Hoang Tran.

**Project administration:** Huong Thi Tran, Trinh To Tran.

**Software:** Huong Thi Tran, Trinh To Tran.

**Supervision:** Minh Quang Nguyen.

**Visualization:** Hung Tien Nguyen, Huong Thi Tran, Trinh To Tran.

**Writing – original draft:** Minh Quang Nguyen, Minh Phuong Tang, Trinh To Tran.

**Writing – review & editing:** Minh Quang Nguyen, Nguyen Binh Minh Hoang Tran.
